# Development of Commercial Health Insurance in China: A Systematic Literature Review

**DOI:** 10.1155/2018/3163746

**Published:** 2018-06-28

**Authors:** Weng I. Choi, Honghao Shi, Ying Bian, Hao Hu

**Affiliations:** State Key Laboratory of Quality Research in Chinese Medicine, Institute of Chinese Medical Sciences, University of Macau, Macau

## Abstract

Facing difficulties like increasing health burden and health inequity, China government started to promote commercial health insurance (CHI) in recent decades. Several policies and announcement have been issued to build up a favorable environment for development of commercial health insurance. Meanwhile, scholar tried to investigate the related issues in purpose to further improve the situation in China. Therefore, we performed this systematic review in order depict a comprehensive picture on the current evidence-based researches of CHI in China. We searched PubMed, ScienceDirect, and CNKI, supplemented with hand search in reference lists, for eligible studies published from 1990 January to 2018 April. Also, hand search was conducted to select suitable articles from international organization and reference list of eligible articles. Two independent reviewers extracted the data from eligible articles and input into a standardized form. Based on the inclusion criteria, 35 articles were included in this systematic review. Most of the studies were quantitative researches with topics such as the development level of commercial health insurance in China, the demand and supply issues related, and the relationship and influence of social health insurance, as well as the moral issues evolved from commercial health insurance system. In summary, CHI in China is still at the early development stage. Among those few evidence-based articles, the findings suggested several policy implication and different market strategy. With the initiation of new health reforms and implementation of taxes policy, more empirical researches should be conducted on issues relating to the practical operation of CHI.

## 1. Introduction

Since last century, with changes in socioeconomic structures, demand on healthcare services and healthcare expenditure level has dramatically arisen. Meanwhile, the enlarging gap between the rich and the poor leads to health inequity due to modernization and economic boom. Being one of the Brazil, Russia, India, and China (BRICs) members, China government is facing several difficulties leading to these problems. Obviously, regarding aging population, the situation is worsening in China because of its largest population in the world. In 2015, the total population was already 1.4 billion [1]. According to World Population Ageing 2013, China was classified as one of the countries with aging population and in forecast, those aged 80 years or over will be 90 million in 2050 [2]. Apart from this, with the influence of social two-child policy announced in 2016, it indicates that the demand for health services, especially for maternal and pediatric care, will be in boom in the coming several years. Besides, the increasing rate of total health expenditure (13.61%) was higher than that of GDP (9.65%) [3]. As a result, it is believed that the continuous increasing trend on total health expenditure would be unchanged in near future. In addition, another problem is the unequal accessibility and service utilization of health services between rural and urban and between the rich and the poor [4]. In order to relieve these healthcare problems, central government has launched and reformed several social health insurance schemes in last few decades. To date, there are three main schemes covering different population in rural and urban separately, including Urban Employee Basic Medical Insurance (UEBMI, launched in 1998), New Cooperative Medical Scheme (NCMS, launched in 2003), and Urban Resident Basic Medical Insurance (URBMI, launched in 2007) [5–7]. These schemes aim to provide universal coverage of health services and alleviate the unbalanced accessibility of medical services between different social classes. Until the end of 2015, around 1.3 trillion Chinese citizens were covered by social health insurance, accounting for 96.5% of total population [8]. Despite its high coverage, limitation on medication, type of medical services, health organization, and reimbursement rate restrict the utilization and protective effect of social health insurance [9, 10]. In such situation, the role of commercial health insurance (CHI) becomes more important.

As a market-oriented health financing tool, literature has shown the effectiveness of CHI in dealing with many healthcare system challenges. Therefore, many countries started shift to public-private partnership model to share the burden among different parties, so as the Chinese government. Although China started market reforms in the 1980s, the coverage of CHI was low after the reforms. CHI is considered as the complementary coverage for those uninsured persons/services/expense by governmental insurance. There were more than 100 insurance companies providing CHI services, ranging from medical insurance, sickness insurance, long-term care insurance, and disability insurance in 2014 [3]. Meanwhile, the total insurance premium of CHI reached 158 trillion, with growing rate of 41.3% [3]. Regardless of this impressive development, CHI was only 3.8% of the total national health expense [4]. This revealed that there are still many improvement areas for CHI market in contributing to health financing system in China. Therefore, Chinese government started to promote CHI. From 2009 to 2015, several opinions were issued regarding health insurance. In 2009, ‘Opinions of the CPC Central Committee and the State Council on Deepening the Reform of the Medical and Health Care System' has clearly clarified the importance of CHI and encouraged enterprises and individuals to actively participate in CHI. Later in 2015, ‘Implementing the Pilot Program of Individual Income Tax Policies for CHI' was issued and came into effect on 1 January 2016. Under this program, Beijing and other 30 cities are designed as the pilot areas. People in these areas are eligible for individual income tax deduction for purchased qualifying CHI.

To be noted, the health care system and health insurance in Hong Kong and Macao are different from the one in China in various aspect. Along the time, government in both regions does not provide social health insurance for public, which is a major difference compared to China. Instead, health voucher system has been launched in 2009 in both regions, but the target population is different: the elderly aged 65 or above is eligible for receiving HKD 2,000 voucher each year in Hong Kong while holder of Macao Permanent Resident Identity Card is eligible for receiving MOP 600 each year in Macao [11, 12]. Meanwhile, colonial background and the notion of health insurance were well spread and high awareness was resulted among public. This is favorable for the development of CHI in Hong Kong and Macao. According to Thematic Household Survey (THS) in Hong Kong, almost 3.3 million persons (91.3% of total population) were covered by commercial health insurance in 2016 [13]. Therefore, due to the uniqueness on CHI development and demographic background in these two regions, this paper only focuses on CHI in China.

In the academic area, scholars started to investigate the insurance system in China. When looking at the foreign studies regarding CHI, researchers have been deeply investigated related issues comprehensively since a long time ago. Comparatively, with the late CHI development in China, CHI-related literatures were mainly based on personal opinions and experience or case study from other countries with well-developed insurance systems. Though most of the articles aimed at giving suggestions like policy enforcement, the reliability of these suggestions was low due to lack of scientific evidence. Simply said, most of them were descriptive articles and seldom consolidated with empirical data. Despite the limited evidence, there is no review presenting and summarizing the findings and their implication. Consequently, it is hard to identify the current knowledge gap and so the future research direction.

Therefore, we performed this systematic review in order depict a comprehensive picture on the current evidence-based researches of CHI in China. In detail, we summarized the topics that were investigated and the corresponding findings. From their implications, practical suggestions and future research direction could be concluded.

## 2. Methods

### 2.1. Search Strategies

To obtain most of the available articles, three various approaches were adopted for searching process. To begin with, search was conducted in four major databases including PubMed, ScienceDirect, Web of Science, and CNKI. Words such as “China/Chinese”, “Commercial/Private”, “Health/Medical”, and “Insurance” were used as the search terms. Searching with different combination of the words could maximize the number of articles resulted, for example, “China AND Commercial AND Health AND Insurance”, “Chinese AND Commercial AND Health AND Insurance”, and “China AND Private AND Health AND Insurance”. Apart from the database search, reference lists of the retrieved articles were reviewed for additional articles. Furthermore, since some of the related researches are conducted outside the academic circles, hand searching was made by using the same keywords in the webpages of international organizations like World Bank, World Health Organization, and China International Conference on Insurance and Risk Management. All the literature on or before 2018 April was searched, for eligible studies published from 1990 January to 2018 April.

### 2.2. Study Selection

After performing the actions according to the search strategy, a large quantity of articles was found with duplication being removed. In order to shortlist the resulting articles, title and abstract were reviewed for simple initial screening. Those targeting social/community insurance and nonhealth insurance were excluded. Regarding the study design, systematic review was rejected. Then, a full-text review was done based on the following inclusion criteria, which were considered as the principle for study selection. By conducting the mentioned screening process, 35 articles were selected for this review. The inclusion criteria included the following:

- Object of study: commercial/private health/medical insurance.
- Study design: empirical study.
- (RCT, cohort, case-control study, or cross-sectional studies).
- Data source: primary and secondary data
- (survey, interview, census, report, and journals).
- Outcome: coverage; resource mobilization; quality of care; provider efficiency; moral hazard; financial protection and equity; OOP spending; access to care.
- Publication: academic journal.
- Search period: 1990 January to 2018 April.
- Language: English and Chinese.
- Location: China.
- Population: Chinese.

### 2.3. Data Extraction and Analysis

**Figure 1:**
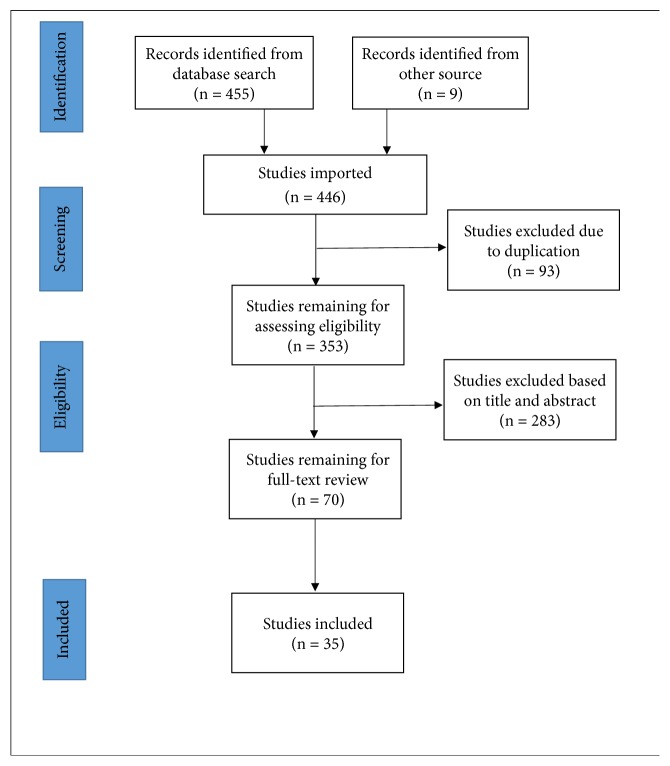
The flowchart of the result searching and selection.

From each article, data was extracted including name of author, publication date, study design, target population, sample size, objective of the study, methodology, analysis, and key findings. Finally, all the information was recorded in standard data extraction table. The whole workflow is summarized as Figure 1.

## 3. Results

In this section, the first part illustrates general characteristic of the reviewed papers. Regarding the objective and measurement of the studies, the result is categorized into several groups, including issues related to demand and supply, development level of commercial health insurance, health financial protection functioning, social health insurance, and other issues.

### 3.1. Characteristic of the Studies

Out of the 35 reviewed papers, around one-fifth were reported in English while the rest were written in Chinese, in the period from 2007 to 2018. The following are the highlights with regard to the study design and methodology. First, most of the researches were in quantitative design; except one study included both quantitative and qualitative measurement [14]. Secondly, two-thirds of the studies investigated the situation of CHI in China as a whole and several studies targets on exploring the provincial level in various areas, such as Beijing, Heilongjiang, and Nanning. With different study area, the former group utilized data from China Health and Nutrition Survey (CHNS) and other national panel data. In the other side, survey was adopted by other researchers for investigation of individual provinces. Therefore, due to different data sources, the sample size is ranging from several hundreds to over 10 thousand. Thirdly, regression analysis has been used predominantly in the reviewed paper.  Table 1 listed the basic characteristic of 35 reviewed papers, including language, study design, source of data, target population/sample size, and the reference of the reviewed paper with publication year.  Table 2 reported the main findings of the reviewed papers.

### 3.2. Current Development Level

To understand current CHI development in China, researchers used CHI premium (in total or per person) as an indicator of development level [15–17]. In comparison along the time, CHI development remained the same from 2005 to 2010 [18]. This matches with the result from another study: CHI coverage did not drop significantly under the enlarged coverage of social health insurance by comparing the data in 2006 and 2009 [19]. In the latest study released in 2018, total CHI premium in 2015 was 5 times more than the one in 2006, which indicated the rapid CHI development in last few years [17]. Furthermore, several findings revealed that there was a serious regional imbalanced CHI development and identified several development conditions in China [17, 18, 20]. In overall, eastern region contributed more than 50% of total premium while western region only contributed less than 20% despite its largest increase from 2006 to 2015. National statistics also revealed this difference: CHI premium in Beijing was around 5.7 billion dollars in 2009 while in Tibet the premium was only 17.7 million dollars [21]. Regarding the current situation, Beijing and Shanghai were cities with most developed CHI system among all cities [16, 18, 22]. Tianjin and Zhejiang were with relatively better development but at the stage of ‘bottleneck'. On the other side, due to slow economic development, low development level resulted in some provinces like Jiangxi, Henan, and Hubei [16, 18, 22]. To be noted, the differences in CHI premium between provinces were even increasing, which increased from 269 yuan in 2005 to 1,072 yuan in 2015 [17]. This indicated that the development gap between the provinces is worsening.

In addition, factors affecting CHI development have been explored. Taking income and insurance awareness as an example, increase in both factors can significantly encourage CHI development [15, 21, 23, 24]. Also, the effect of proportion of elderly is ambiguous. It was not significant in improving CHI development in two studies targeting urban population while in one study conducted in Anhui Province it was one of determinants of CHI demand [15, 23, 25, 26]. Apart from these demographic factors, availability of medical resource was positively associated with CHI development [23]. Another medical related factor, health expenditure, the findings were ambiguous. With 31 provinces data, the study illustrated that health expenditure per capital had no significant effect on CHI development [15]. Later in 2012, by using telephone survey in Beijing, Shanghai and Xiamen, commercial insurance coverage was significantly associated with medical expense [27].

One of the special findings has been demonstrated in a study conducted in 2011. With investigation on insurance market structure, the result suggested that market competition could improve CHI development. Another finding indicated that both participation of foreign and professional health insurance company was insignificant in improving CHI development [28]. Regarding the influence of social health insurance, it would be illustrated in the next section.

### 3.3. Demand and Supply

As CHI development in China is still at early stage, there is potential for coverage improvement [29]. Due to multiple factors, the enormous potential demand on CHI were underestimated, especially among urban population [30, 31]. In one study conducted in Sichuan and Shandong, findings revealed an estimation of annual premium of more than 20 billion RMB within a small city. Therefore, many studies targeted on investigating the consumer purchasing behavior in order to explore the potential market. Out of 35 articles, the related topic has been explored in almost one-third of the reviewed papers. The purchase of CHI always represented as demand indicator, and in order to quantify it, CHI premium in total or per capital was adopted as measurement [14, 24–26].

A trend was shown that, as the age increased, the take-up rate of CHI was higher [30, 32]. Moreover, there is evidence showing that socioeconomic status was significantly associated with demand on CHI. For example, population with higher income, family saving, and educational level was more likely to buy CHI [14, 24, 29, 33]. Also, people working in private enterprises or being self-employed were more willing to buy CHI than those working in governmental organization [14]. Regarding medical related factors, the higher the health expenditure, the higher the demand on the CHI [21, 26, 34]. Furthermore, other indicators like insurance awareness were significant in increasing CHI demand [14, 35]. On the other side, the level of CHI premium was positively related to the educational level but education expenditure had no significant effect on demand [20, 26, 36].

Researchers also focused on the varied CHI demand in different population and regions in China. The CHI demand of elderly was higher than other populations [35]. With better health status, higher education level and unstable marital relationship, elderly are more likely to purchase CHI [37]. Among elderly male was more willing to buy CHI than female [37]. Also, families with more elderly members showed greater willingness in insurance participation [21, 24, 38]. On the other side, under the participation of social health insurance, male was more willing to buy CHI than female. Without joining social health insurance, the situation changed, in which female was more willing to buy CHI than male [39]. When looking at the single effect of participation of social health insurance, participants of social health insurance were less likely to purchase CHI [38]. Besides, same factor can have different effect in various geographic location in China. Change in health expenditure per person and rural family income per person can lead to significant change on CHI demand in eastern regions [33]. For western regions, only rural family income per person can lead to significant change on CHI demand. However, in central regions, change in health expenditure per person and rural family income per person did not lead any significant change on CHI demand [33]. Similarly, in another study CHI demand determinant was different among Beijing, Shanghai, and Hubei [16]. Number of social health insurance participant was positively related to CHI premium in all three regions. However, family annual health expenditure per urban resident and elderly dependency rate were positively related to CHI premium in Beijing and Shanghai, respectively, while it was negatively related in Hubei.

Household registration system in China classified Chinese citizen into two groups: urban and rural resident. Due to the slow CHI development in rural area, the sampling population of several reviewed studies are urban population [15, 16, 21, 23, 24, 28, 35, 40]. Demand for CHI in urban areas has greater potential than rural area and urban residents are more likely to purchase CHI than rural residents [14, 32, 38]. Due to the difference in socioeconomic background, the influential factors of CHI demand are different between two groups. For urban family, younger and married householder is more likely to purchase CHI while rural family with less family burden and younger healthy householder is more likely to purchase CHI [40]. Besides, the effect of education level on CHI demand is different. Higher educational level could increase the purchase of CHI in urban family but reduce in rural family. [40]. One reviewed study investigated the effect of social networking on the purchase of CHI. Independent variable indicating social networking was the number of relative and friend visits, respectively, during Chinese New Year. Findings were different among urban and rural population. In the eastern and middle rural regions of China, social network had a significant positive effect. On the contrary, there was no significant effect on rural people living in the central and western region and all urban people [41].

Apart from these, specific comparison of demand on various insurance was conducted. In a survey research conducted in Sichuan and Shandong, willingness-to-pay for three different kind of health insurance was compared. Findings demonstrated that individual was more likely to buy and pay more premium for major catastrophic disease insurance (MCDI) and inpatient expenses insurance (IEI) than outpatient expenses insurance (OEI) [14]. Another determinant, social health insurance, can drive the development of CHI with saving purpose but no effect on health protection-oriented CHI [42].

In the point of view of insurance company, topics related to level of premium and claim cost were investigated. Among the reviewed paper, there are two articles investigating the influential factors of claim cost. In the one focusing on inpatient claim cost, two findings have been concluded [43]. Firstly, the effect of inpatient health expenditure on claim cost was significant but not as high as expected. Secondly, the number of A&E patient admitted to inpatient department had great effect on claim cost. Another study illustrated that claim cost could be affected by several factors, resulting in the individual difference in premium. Obviously, the claim cost increased with the increase in age and the number of hospital day. Besides, gender, marital status, occupation, and the origin of patient were found to be associated with claim cost [44].

### 3.4. Social Health Insurance

The implementation of New Cooperative Medical Scheme (NCMS) in 2003 brought changes to the health insurance systems and so as to CHI system. There is no doubt that the development of social health insurance is better than CHI and it plays more important role than in China nowadays [18, 20]. With social health insurance as the main sector, many researches confirmed the complementary role of CHI, as the initial insurance market structure was already formed [30]. Nevertheless, there is still area of improvement for CHI development and the cooperation of social health insurance and CHI [18, 29].

Whether the influence of social health insurance is positive or negative to CHI was controversial. Some studies indicated that social health insurance could boost CHI development or demand [21, 22, 28, 29]. On the contrary, coverage level of social health insurance was found to be inversely correlated with CHI development in several studies [15]. In a study conducted in 2016, decrease in the demand of CHI was associated with social insurance coverage, especially for urban residents who were covered by URBMI [38]. Similar result has been found in the previous study conducted in nine cities in 2012 and later study conducted in 2018 [24, 45].

As in China social health insurance is classified by household registration, urban residents enroll for UEBMI and URBMI and rural resident enroll for NCMS. Therefore, some studies investigated the effect of each type of social insurance on CHI development separately. Based on a study comparing the situation in 2004, 2006, and 2009, the relationship between NCMS and CHI at first was as substitute to each other and later became complementary [32]. Later in 2011, evidence proved the complementary role of CHI as adults were 2.1 % more likely to purchase CHI when NCMS became available [44]. Among rural residents, people with increasing age, chronic diseases, lower family income, large-size family, smoking habit, and without preventive care are more unlikely to purchase CHI [32]. On the other sides, the influential factors are different between UEBMI and URBMI. For UEBMI enrollee, increase in UEBMI premium can increase CHI premium while increase in disposable income was the prominent factor for URBMI enrollee [32]. Also, UEBMI was found to have promotion effect on CHI development while there was no significant effect resulting from URBMI [24].

Specially, one study explored the degree of coordination of social health insurance and CHI. Researchers defined the depth and density as key indicators for representing the coverage of CHI and social health insurance. The coordination was not high in overall and improved during 2005 to 2010, though the improvement is not significant. By comparing the situation in different provinces, it showed that coordination level was related to economic development level [18]. Furthermore, the cooperation of CHI and social health insurance was associated with health financial protection level.

### 3.5. Health Financial Protection and Equity Functioning

The effectiveness of CHI in health financial protection has been evaluated in several studies. The findings indicated that CHI brought positive effect in health financing protection [22, 27]. However, it only played a minor role, compared to the enormous coverage of social health insurance in China [46]. Some researchers developed model with different variables contributing to health protection level, for example, demographic, medical, and insurance related indicators. Both depth and density of CHI can improve the protection level while the CHI premium was negatively related to the protection level [20]. In addition, the effect of CHI on financial protection level varied among regions [20, 22]. With the most developed commercial health system, health financing protection level was highest in Beijing and Shanghai. In some provinces such as Hebei, Shanxi, and Heilongjiang, the elasticity of demand on CHI was not high, showing that population in those areas were highly relied on social health insurance and out-of-pocket payment, resulting in a lower financial protection level. In another study, CHI demand elasticity varied in terms of geographic location. It was low regarding population and national GDP per person in eastern regions. For western regions, CHI demand elasticity was high regarding population but low regarding national GDP per person. However, in central regions, CHI demand elasticity was high regarding population and national GDP per person.

In terms of types of service, CHI was more effective in reducing out-of-pocket payment in preventive health service, compared to treatment services [19]. To be concern, the issue of moral behavior caused by CHI resulted in increasing the out-of-pocket payment proportion of medical care expenditure, though studies showed that CHI can significantly reduce the self-payment of medical expenditure [19]. Besides, the equity of current health financial protection in China was not well-distributed and the finances of CHI was progressive among the rich and poor [34, 46]. Later result also showed that the alleviation function of CHI on inpatient and outpatient expenditure was insignificant among low income people [19]. Furthermore, the financing of CHI varied in urban and rural. The financing performance was in proportional in urban while it was regressive in rural. Overall, the performance in rural area was superior in rural area [34, 46].

### 3.6. Other Issues

Beside the common topics mentioned, consumer satisfaction was examined. Gender is one of the significant factors related and findings showed that women were more satisfied than men. Also, people with higher education level and lower level of CHI understanding were more satisfied [47]. Moreover, moral issues related to public, insurance company, and medical sides have been discussed. Firstly, on the issue of risk selection, the strategies adopted by insurance companies were different in urban and rural area. For urban residents, the selection mainly depended on one's age while it was based on the place of residence for rural residents [30]. Secondly, both urban and rural residents have significant adverse selection behavior [30]. Urban residents with chronic disease were more likely to purchase commercial insurance while rural residents being overweight or underweight were more likely to purchase. In addition, intangible health resource like health professional could reduce the health financing protection level [20]. As CHI could help to reduce the proportion out-of-pocket payment, the medical expenses were increased by the immoral health professional for maximizing the profit. This resulted in increase in the out-of-pocket payment at the end, and therefore protection level was even worse before purchasing the CHI.

## 4. Discussion

This paper has reviewed literatures investigating CHI in China. In the light of evidence illustrated in this review, several findings should be interpreted comprehensively for the implication hindered.

Currently in China, CHI development was lagging behind social health insurance. The following are main reasons concluded from the reviewed papers. Firstly, due to cultural background, the risk attitude of Chinese population tends to be postevent control type [35, 48]. As insurance is considered as a preevent protection tool, the acceptability and insurance awareness are not high in Chinese community. In a survey conducted in Nanning city, the respondents did not fully understand CHI, including the coverage, liability, and additional products [31]. It reflected that, in Chinese society, knowledge level about CHI was low. This highly prohibited CHI development. Secondly, with universal coverage of social health insurance, more people start to think of health financing protection. However, misconception of CHI still existed [31]. For example, people could not clearly distinguish the role and function of CHI and social health insurance, resulting in thinking that it is not necessary to have extra insurance when they were covered by social health insurance. Subsequently, this potential demand was not effectively transform to a real purchasing power on CHI. Thirdly, even when people have the correct perception, the situation became as follows: either no suitable products and services were provided or people were rejected due their demographic characteristic, such as elderly. Therefore, as CHI could not meet their needs, people would turn to rely on other health financing protection tools. Fourthly, CHI was inefficient in alleviating the pressure of heavy health expenditure on Chinese patients [19]. The financing situation of patients with insurance was even worse as the proportion of out-of-pocket payment was increased resulted from the moral issues. Comparatively, public tended to choose social health insurance instead of CHI in the perspective of actual health financing protection level. Fifthly, in the individual level, high premium, participation of social health insurance and complex design of insurance products and claim process were the obstacles for purchase CHI [31]. Apart from these, with respect to development variation among the provinces, the situation was led by the diverse economic development level and purchasing behavior. Due to the unequal allocation of medical resource among regions, the variety and accessibility of healthcare services were relatively lower in some places. With such insufficient support of medical services, it is not favorable for CHI development [48].

From one of the reviewed paper, it is stated that there was unbalanced CHI development between eastern and western regions and also within eastern and western regions, in which the difference was more prominent within the regions [17]. The household registration system could be one of the potential leading causes. Due to superior situation on financial support, accessibility to social resources, and enrollment of different social health insurance (NCMS and UEBMI/URBMI), urban residents are more likely to purchase CHI, which is favorable for CHI development among urban cities [14, 32, 38]. This resulted in more rapid CHI development and higher CHI coverage level in urban than rural areas. Several studies explored the different demand determinant and purchase behavior between these two populations. However, in one study, the data illustrated that rural residents are more likely to purchase CHI due to the lower health protection level provided by NCMS [37], in which the evidence is not consistent with other studies. As there are more papers which examined the CHI development or related issue in urban population, more consolidate evidence about rural population should be provided. Therefore, apart from the leading causes of this uneven development, the difference between urban and rural population relating to CHI should be addressed in future research for filling the knowledge gap in this aspect.

Having identified the obstacles and challenges facing, researchers aimed to give evidence-based suggestion on promoting CHI by investigating influential factors. Generally, age, disposable income, and educational level were the key determinants on decision of purchase CHI [30, 40]. Among these factors, several findings were highlighted. Findings regarding elderly were contradicted in some extent [15]. On one side, there was great potential of insurance market among the elderly. In other studies, the proportion of elderly was not a significant force on pushing CHI development. Towards such result, researchers argued that the enormous proportion of elderly was in rural area, whose affordability was relatively lower than urban elderly. This resulted in a phenomenon that rural elderly was in need to purchase CHI but cannot transform to a real demand. Another situation is that even the urban elderly was capable of paying for insurance premium, the insurance companies neither provided suitable products nor adopt risk selection to reject them. In terms of cultural factor, in Chinese society, CHI is not regarded as the way of protection from health risk as in traditional norms elderly rely on their children for handling the medical needs [23]. Furthermore, in normal situation with increased income, people were more willing to purchase CHI. However, when the income reached certain level, people considered that the health protection capability was high enough without relying on CHI [16, 31]. Other factors like insurance awareness has been proven to be significantly associated with CHI demand. Meanwhile, the supply by insurance company was not sufficient [30], especially in the types of products. This restricted CHI development in some places resulted in bottleneck stage. Surprisingly, the participation of foreign insurance company did not have any effect on encouraging the competition. This could be explained by current small market proportion and its conservative attitudes on development in China.

These researches have provided scientific evidence for insurance company on design and marketing strategy of insurance products. Findings indicated that CHI company cannot solely rely on the basic insurance products. More comprehensive products or personalize service should be developed as people became more aware on health issues. Similarly, due to the various characteristic in different region, insurance company should have different strategies and products in order to meet most of the health needs. For example, different risk preference between male and female and participation of social health insurance should be taken into account [39]. Besides, the current premium level was revealed as much higher than the expected one due to too simple estimation [44]. Insurance companies should take the related factors, such as marital status and occupation, into account for more comprehensive estimation of premium, avoiding overpricing on premium level.

The effect of social health insurance towards CHI system was controversial [23, 28, 38]. In the positive side, the initiation of social health insurance has raised public awareness on health burden and insurance. Also, despite the high coverage rate of social health insurance, the protection level and scope are low and social health insurance is reimbursement type. Therefore, there are still rooms for CHI to expand by providing supplementary insurance products. Furthermore, Chinese government is active in promoting the supplementary role of CHI in the whole insurance system. The environment became even more favorable for CHI development since new healthcare reforms in 2009. On the contrary, others argued that, by the expansion of social health insurance, the demand on health protection could be fulfilled, resulting in reduced need on CHI. Subsequently, the influence of social health insurance largely depended on the role of CHI taken in the whole insurance. Moreover, across the time from 2005 to 2010, the coordination between CHI and social health insurance have improved but the effect was not significant. This was mainly attributed by the inconsistent development rate of social health insurance and CHI. This implied that desirable development of insurance system in China could be achieved by the well-balanced development of both insurances.

Due to the information asymmetry, risk selection was adopted by insurance company to avoid accepting too many high-risk participants. In China, the situation did happen and the key determinant of selection varied between urban and rural [30]. Insurance company is based on the age of urban applicant for risk selection while in rural area, it is based on the resident area of the applicant. Even the company applied sales restriction on certain rural area with less medical resource in order to avoid applicant with worse financial situation. In consumer side, adverse selection occurred as the households in worse health conditions more likely to purchase commercial insurance [27]. Moral issues happened between other parties as well, for example, medical professionals and insured population. Under the initiation of new health reforms, different medical moral problems including drug rebates, overprescription, and unreasonable increase in medical expense were resulted. These problems have already restricted the health financial protection functioning of health commercial insurance [14].

Several limitations should be addressed hereby. First, this systematic review only included a small number of articles with limited findings. Although a comprehensive search strategy was conducted, only 35 articles with empirical data were selected. In some of issues such as moral issues, only few articles mentioned and limited the generalizability of the findings. Second, regarding the methodological quality, several reviewed papers did not provide details of study design, for example, number of samples and sampling methods. This may affect the validity and reliability of the measures. Third, due to the wide range of topics and limited evidence, effect synthesis was not done for statistical power of the findings. Through the intensive summary of past researches, this systematic review gives some research direction in the related field. Firstly, from the reviewed paper, there is no research which examined the influence of tax policies on encouraging CHI development in China. Similar researches have been done in many different foreign countries like Australia and European [49]. With the newly implemented individual income tax policies pilot program in 2016, it is crucial to evaluate for effectiveness of tax policy in order to fill the knowledge gap and provide scientific evidence in this aspect. Secondly, regional difference was illustrated on the development of CHI and health needs of consumers. Also, the findings of a large portion of evidence-based articles were concluded from analyzing the national data. This implied that it is essential to individually explore at provincial level so that more specified and practical suggestions could be given to government and insurance company at that area. Thirdly, several parties were involved in the whole insurance systems. However, investigation on the supply perspective (insurance company) and medical perspective (health professional and organization) should be continued as insufficient information was provided at current stage. Finally, from the review findings, health insurance education should be provided for public as misunderstanding was one of the obstacles existing in China now. However, we lacked education details such as content and channel. Further exploration on public knowledge gap in this aspect was certainly needed.

## 5. Conclusion

In summary, CHI in China is still at the early development stage compared to other developed countries. Because of this, literatures were mainly based on the authors own opinion or experience. Among those few evidence-based articles, topics such as current development level, influential factors within the systems, relationship with social health insurance, and other issues have been addressed. From these findings, policy implication and different market strategy were provided. With the initiation of new health reforms and implementation of taxes policy, more empirical researches should be conducted on issues relating to the practical operation of CHI.

## Figures and Tables

**Table 1:** Basic characteristic summary of the reviewed papers.

**No.**	**Language**	**Study Location**	**Study Design**	**Data Source**	**Target population /Sample size**	**Author and Publication year**

1	English	Sichuan and Shandong	Quantitative and qualitative	(a) Household survey (b) Interview	2,630 households (Survey) 550 households (Interview)	Ying XH et al. (2007)

2	English	Guangxi, Guizhou, Heilongjiang, Henan, Hubei, Hunan, Jiangsu, Liaoning, and Shandong	Quantitative	2000, 2004, 2006 China Health and Nutrition Survey (CHNS)	Rural residents 17,716 adult sample 3,079 child sample	Liu H, Gao S, Rizzo JA (2009)

3	Chinese	31 Provinces	Quantitative	1998 to 2004 national panel data	31 Provinces	Shao QQ, Chen J (2009)

4	Chinese	Hubei, Beijing and Shanghai	Quantitative	2002 to 2007 statistic	Hubei, Beijing and Shanghai	Li Q (2009)

5	Chinese	31 Provinces	Quantitative	1999 to 2007 national panel data	31 Provinces	Lin QZ (2010)

6	Chinese	31 Provinces	Quantitative	1998 to 2009 national panel data	31 Provinces	Liu FF, Wang XH, Bian H (2010)

7	Chinese	31 Provinces	Quantitative	2002 to 2009 national panel data	31 Provinces	Wang XN (2011)

8	Chinese	31 Provinces	Quantitative	2005 to 2009 national panel data	31 Provinces	Li HL (2011)

9	Chinese	31 Provinces	Quantitative	1997 to 2009 national data	31 Provinces	Li BR(2011)

10	English	Beijing, Shanghai and Xiamen	Quantitative	Telephone Survey	5,097 households	Fang K, Shia B, Ma S (2012)

11	English	Gansu	Quantitative	Household survey local statistics yearbook Interview	Year 2003:3946 households (13,619 individuals) Year 2008: 3958 households (12,973 individuals)	Chen M, Chen W, Zhao Y (2012)

12	Chinese	31 Provinces	Quantitative	1985 to 2010 national panel data	31 Provinces	Wu HB (2012)

13	Chinese	31 Provinces	Quantitative	2000, 2004 and 2006 CHNS data	City sample: 8,402 Rural sample: 16,645	Liu H, Wang J (2012)

14	Chinese	China	Quantitative	(1) Insurance company (2) Hospital	(1) General hospital population (2) Specific hospital population (3) Social health insurance population (4) Commercial health insurance population	Qiu CJ, Chen T (2012)

15	Chinese	Beijing	Quantitative	Survey	500 persons (i) 233 male and 267 female	Chai XH, Ji CH (2012)

16	Chinese	China	Quantitative	2006 Longitudinal survey data	1,973 participants aged 60 or above	Qiu Y (2012)

17	English	Nanning, Guangxi	Quantitative	Survey	178 respondents	Ye AZ, Lu F, Huang WH, Liang J (2013)

18	Chinese	31 Provinces	Quantitative	2005 to 2010	31 Provinces	Zheng RM, Hua J (2013)

19	Chinese	31 Provinces	Quantitative	2004, 2006 and 2009 CHNS data	31 Provinces	Xu R, Zhang D & Ji X (2013)

20	English	Heilongjiang	Quantitative	Household survey Local statistics yearbook Interview	Year 2003: 3,841 householders (11,572 individuals) Year 2008: 5,530 householders (15,817 individuals)	Chen M, Zhao Y, Si L (2014)

21	Chinese	31 Provinces	Quantitative	2003 to 2012 panel data	31 Provinces	Zhu ML, Gui ZX (2014)

22	Chinese	31 Provinces	Quantitative	1999 to 2011 panel data	31 Provinces	Liu JY (2014)

23	Chinese	China	Quantitative	1997 to 2011 panel data	31 Provinces	Liu R & Liu HX (2014)

24	Chinese	31 Provinces	Quantitative	2004 to 2013 panel data	31 Provinces	Suo LY, Wanyan RY, Chen T (2015)

25	Chinese	31 Provinces	Quantitative	2000, 2004, 2006 and 2009 China Health and Nutrition Survey (CHNS)	31 Provinces	Jiao N (2015)

26	Chinese	China	Quantitative	2011 panel data	31 Provinces	Liu HL (2015)

27	English	China	Quantitative	Survey	17,705 respondents	Jin Y, Hou Z, Zhang D (2016)

28	Chinese	China	Quantitative	Survey	Rural Sample: 7,403 Urban Sample: 6,072	Zhou X, Sun J (2016)

29	Chinese	Nanning City	Quantitative	Survey	263 sample	Li HF (2016)

30	Chinese	25 Provinces	Quantitative	2011 China Household Finance Survey	Total sample: 5,295 Rural sample: 2,893 Non-rural sample: 2,402	Fu YZ & Su ZF (2016)

31	Chinese	China	Quantitative	2006 to 2015 Premium income database	31 Provinces	Zhang ML et al. (2018)

32	Chinese	China	Quantitative	2009 to 2015 panel data	31 Provinces	Ni L & Feng GZ (2018)

33	Chinese	Anhui Province	Quantitative	2002 to 2015 statistics yearbook and insurance statistic report	Anhui Province	Zhao HF (2018)

34	Chinese	China	Quantitative	2004 to 2014 panel data	31 Provinces	Yan JJ (2018)

35	Chinese	China	Quantitative	1997 to 2015	31 Provinces	Zhu JM & Wu ZH (2018)

**Table 2:** Summary of measurement variable and main findings of the reviewed papers.

**No.**	**Dependent variable**	**Independent variable**	**Findings**

1	Willingness-to-buy	(1) CHI premium (2) Public health insurance coverage (3) Age (4) Employment, educational and health status (5) Income	(1) The demand for CHI in urban areas had great potential. (2) Individuals was more likely to buy and pay more for MCDI and IEI than OEI. (3) Determinants of CHI demand were similar in the three programs (employed by private enterprises or self-employed, aged under 40, college educated, and higher income).

2	Purchase of CHI	(1) New Cooperative Medical Scheme (NCMS)	(1) Adults were 2.1 % more likely to purchase CHI when NCMS became available. (2) NCMS had a larger positive effect on adult private coverage in higher income groups and in communities with CMS. (3) For adults and children, risk preferences and socio-economic status are important predictors of commercial insurance take-up. (4) No evidence for adverse selection in the demand for private health insurance.

3	Health Protection Level	(1) Provincial GDP (2) Number of hospital bed per 1000 persons (3) Number of doctors per 1000 persons (4) Depth and density, premium of CHI	(1) GDP, coverage level of social health insurance, number of hospital bed per 1000 persons, depth of CHI, density of CHI was positively related to the health protection level. (2) Number of doctors per 1000 persons and premium of CHI was negatively related to the health protection level. (3) Tangible healthcare resource boosted the healthcare efficiency but the intangible one like doctors reduced due to moral issues. (4) Cooperation of CHI and Social health insurance was good in the region with high health protection level.

4	CHI premium	(1) Disposable income (2) Family annual health expenditure per urban resident (3) Number of social health insurance participant (4) Social health insurance premium (5) Elderly dependency rate	(1) CHI development level is higher in Beijing and Shanghai than in Hubei. (2) In Hubei region, CHI premium is positively related to disposable income, number of social health insurance participant but negatively related to family annual health expenditure per urban resident and elderly dependency rate. (3) In Shanghai region, CHI premium is positively related to number of social health insurance participant and elderly dependency rate. (4) In Beijing region, CHI premium is positively related to disposable income, number of social health insurance participant, social health insurance premium and family annual health expenditure per urban resident.

5	Health expenditure per person	CHI premium per person - indicator of level of CHI development	(1) CHI could promote the health protection level. (2) The effect of CHI in promoting health protection level is different among regions.

6	Total CHI premium	(1) Income per person (2) Health expenditure per person (3) Percentage of governmental health expenditure (4) Percentage of elderly population (5) Total population: resource level (6) Depth of CHI	(1) Disposable income and insurance awareness significantly promote the development of CHI (2) Level of social health insurance is inversely correlated to the development of CHI. (3) Health expenditure per persons, total population and the proportion of elderly have no significant effect on development level of CHI.

7	CHI premium per person	(1) Social health insurance premium (2) Herfindahl-Hirschman index (3) Proportion of foreign insurance company (4) Health expenditure per person (5) Disposable Income (6) Proportion of elder population (7) Gender (8) Education level	(1) Social health insurance had significantly driven the development of CHI. (2) Market competition contribute to the development of CHI. (3) Both the influence of participation of foreign company and the professional health insurance company in development of CHI is insignificant

8	CHI premium – indicate the demand of CHI	(1) Disposable income (2) Elderly population (3) Insurance awareness (4) Health expenditure	(1) Increase in health expenditure, disposable income and insurance awareness significantly boost the development of CHI (2) The demand of CHI is higher among elderly.

9	CHI premium	(1) Disposable income per person (2) Family health expenditure (3) Number of social health insurance participant (4) Elderly dependency rate	(1) Increase in disposable income, family health expenditure, number of social health insurance participants and elderly dependency rate can promote the CHI demand due to raised awareness on health. (2) There is great difference in different areas in China regarding to the purchasing power of the residents.

10	(1) Insurance coverage (2) Gross medical cost (3) Net out-of-pocket payment	(1) Households (2) Income (3) Medical expense (4) Presence of at least one inpatient treatment (5) Presence of chronic disease, (6) Living in urban/rural areas	Commercial insurance coverage was significantly associated with medical expense.

11	Kakwani index –	(1) Concentration index of health care payments (2) Gini coefficient of gross income	(1) The finances of private health insurance were progressive among the rich and poor. (2) In both cities and villages, the healthcare financing channels of private health insurance and OOP payments were equitable (3) The financing performance of private health insurance in urban areas was inferior to that in rural areas.

12	CHI compensation rate	(1) Number of doctor per 1000 persons (2) Hospital bed occupancy rate (3) Outpatient health expenditure per person (4) Hospital bed per 1000 persons (5) Inpatient health expenditure per person (6) Hospital day	(1) The effect of inpatient health expenditure on the claim cost is significant but not high. (2) The number of A&E patient admitted to inpatient department has great effect on the claim cost.

13	Purchase of CHI	(1) Personal health status (2) Economic status (3) Personal expected healthcare demand (4) Personal risk preference (5) Medical insurance market and policy (6) Gender (7) Marital status (8) Number of family member (9) Age	(1) In urban, insurance company based on the age for risk selection while in rural, insurance company based on the area (2) The potential demand of urban residents is underestimated. (3) People have significant adverse selection behavior both in urban and rural. (4) Personal preference and personal purchasing power affect the demand of insurance (5) Social health insurance boost the development of CHI. (6) The coverage of CHI affects the personal demand.

14	CHI claim cost	(1) Number of hospital day (2) Age (3) Sex (4) Marital status (5) Occupation (6) Origin of patient	All the factors affected the claim cost.

15	Purchase of CHI	(1) Gender (2) Social health insurance	(1) With social health insurance, male is more willing to buy CHI than female. (2) Without social health insurance, female is more willing to buy CHI than male.

16	Purchase of CHI	(1) Demographic characteristic (2) Scio-economic characteristic (3) Health status (4) Behavioral issue (5) Mental state	(1) Male are more likely to purchase CHI than female. (2) With better health status, higher education level and being divorced, elderly are more likely to purchase CHI. (3) Rural residents are more likely to purchase CHI than urban residents.

17	N/A	N/A	(1) The market potential of CHI were not effectively developed (2) There was cognitive biases about CHI among Chinese population. (3) The claims service is mess and the satisfaction is low regarding to the claim process.

18	Degree of coordination	(1) Depth and density of CHI (2) Depth and density of social health insurance	(1) The development of CHI in Beijing and Shanghai is better than the other areas. (2) The development of CHI remains the same from 2005 to 2010. (3) The development of social health insurance is better than that of CHI. (4) The coordination between CHI and social health insurance is not high. It improved but not significant during 2005 to 2010. (5) Coordination level of CHI and social health insurance is related with economic development level.

19	Purchase of CHI	(1) NCMS (2) Family income (3) Age (4) Gender (5) Educational level (6) Marital status (7) Employment status (8) Family size (9) Chronic disease (10) Exercise (11) Preventive care (12) Smoking (13) Family expenditure	(1) The relationship between NCMS and CHI at first is substitute and later be complementary. (2) With increasing age, chronic diseases and large-size family, rural residents are more unlikely to purchase CHI. (3) Increase in family income can lead to increase in CHI demand. (4) Female, people with smoking habit and without preventive care are more unlikely to purchase CHI

20	Kakwani index – indicate the health financing equity	(1) Concentration index of health care payments (2) Gini coefficient of gross income	(1) Healthcare financing distribution in China was unequal. (2) CHI had played a minor role in Chinese healthcare financing due to its low associated coverage

21	CHI Premium	(1) Disposable income (2) Outpatient expenditure (3) Claim proportion of social health insurance (4) Number of medical professional in per 10000 persons (5) Proportion of age over 65 in total population	(1) The increase in disposable income and availability of medical resource significantly boosted the development of CHI. (2) Social health insurance encouraged the development of CHI. (3) The effect of proportion of elderly is not significant.

22	Total CHI premium income	(1) Total GDP (2) Healthcare expenditure price index (3) Medical graduate (4) Total social health insurance premium	(1) With higher education level and depth of social health insurance, premium of CHI increase. (2) The premium of CHI is more sensitive to the increase of education level than to the depth of social health insurance. (3) Medical expenditure price level was not the main cause.

23	CHI premium	(1) Governmental health expenditure (2) Urban and rural family saving	(1) The substitute relationship between social health insurance and CHI is not significant, which means there is still space for CHI development under high coverage of social health insurance in China. (2) With increased family saving, people are more aware about their health and increase in the CHI demand.

24	(1) Health insurance development index (2) Depth of CHI: ratio of insurance premium and local GDP (3) Density of CHI: ratio of insurance premium and local population	(1) Life expectancy (2) Proportion of urban citizen (3) Education level (4) Disposable income (5) Number of health organization (6) Number of health professional (7) Number of hospital bed (8) Distribution of healthcare resource (9) Healthcare expenditure (10) Population density	(1) The development rate of Eastern area was twice of the rate of Middle and Western Area (2) The level of insurance development was not positively related to its economic development. (3) The level of insurance development did not gradually decrease from Eastern to Middle to Western.

25	Out-of-pocket payment	(1) Social health insurance (2) Population (3) Economy status	(1) CHI increases the out-of-pocket payment proportion due to moral risk behavior. (2) The alleviation function of CHI on inpatient and outpatient expenditure is insignificant among low income people. (3) CHI could not satisfy the needs of elderly as most of CHI target customers with age over 60. (4) In 2006/2009, the influence of CHI on the medical expenditure was not significant but CHI can significantly reduce the self-payment of medical expenditure. (5) In 2006/2009, compared to 2000/2004, the coverage of social health insurance was higher but the coverage of CHI did not drop significantly.

26	CHI premium	(1) National GDP (2) Total population (3) Rural family income per person (4) Consumer good index (5) Urban health expenditure per person (6) Number of health organization (7) Geographic area	(1) For eastern regions, CHI demand elasticity is low regarding to population and national GDP per person. (2) For western regions, CHI demand elasticity is high regarding to population but low regarding to national GDP per person. (3) For central regions, CHI demand elasticity is high regarding to population and national GDP per person. (4) For eastern regions, change in health expenditure per person and rural family income per person can lead to significant change on CHI demand. (5) For western regions, only rural family income per person can lead to significant change on CHI demand. (6) For central regions, change in health expenditure per person and rural family income per person did not lead any significant change on CHI demand.

27	(1) Public insurance only (2) Private insurance only (3) Double coverage of public and private health insurance (4) Double coverage of both rural and urban public health insurance (5) No insurance	(1) Policy (2) Health (3) Socioeconomic (4) Risk aversion	(1) Rural residents were more likely to participate in public health insurance than urban residents (2) Rural-to-urban migrants were more likely to be uninsured (3) Large-size families with more elderly members may show greater willingness of insurance participation (4) UEBMI enrollees and high SES population were more likely to buy private insurance (5) Public insurance coverage was associated with a reduced demand for private insurance, especially for urban residents who were covered by URBMI.

28	Purchase of CHI	(1) Number of relative visit during CNY (2) Number of friend visit during CNY (3) Household income (4) Gender (5) Age (6) Martial status (7) Education level (8) Health status (9) Social health insurance (10) Smoking (11) Risk attitudes	(1) Social network has a significant positive effect for rural people purchasing CHI. (2) In the eastern and middle part of the high degree of marketization, there is no obvious effect. (3) In the central and western region, network has significant positive effect for the purchase. (4) Social network is not significant for urban people

29	Consumer satisfaction	(1) Gender (2) Age (3) Marital status (4) Education level (5) Occupation (6) Annual income (7) Understanding of CHI	(1) Gender and education level is significantly related to consumer satisfaction. (2) With higher education level, people are more satisfied with CHI. (3) With deeper understanding of CHI, the satisfaction is lower.

30	Purchase of CHI	Family Burden (1) Family income per person (2) Family property per person (3) Family dependency coefficient	(1) The family with less family burden (higher income, more property and lower family dependency) was more willing to purchase CHI. (2) For urban family, younger and married householder is more likely to purchase CHI. (3) For rural family, family with less family burden and younger healthy householder (4) Higher educational level could increase the purchase of CHI in urban family but reduce in rural family.

31	N/A	N/A	(1) CHI premium increased rapidly but the depth and premium per person were still low compared to other countries. (2) Regarding to national premium income, there was a serious imbalance: Eastern region contributed more than 50% of total premium while western region only contributed less than 20% despite of its largest increase in the past 10 years. (3) The unbalanced CHI development among the areas in Eastern and Western regions was more prominent while in Middle and North-Eastern region the development was even. (4) The differences in premium between provinces was increasing, in which increased from 269 yuan in 2005 to 1,072 yuan in 2015.

32	CHI Income Premium	(1) Disposable income per person (2) UEBMI premium (3) URBMI premium (4) Proportion of general college graduate (5) Elderly dependency ratio (6) Out-patient service fee	(1) Disposable income, UEBMI premium, elderly dependency ratio and outpatient services expenditure were the determinant of CHI demand. (2) For UEBMI enrollee, increase in UEBMI premium can increase CHI premium. (3) For URBMI enrollee, increase in disposable income can increase demand on CHI.

33	CHI premium	(1) Daily expenditure level (2) Personal health expenditure (3) Density of CHI (4) Governmental health expenditure (5) Depth of CHI (6) Elderly population	(1) Daily expenditure level and elderly population can promote CHI demand (2) The current social health insurance system has “Crowding Out Effect” on CHI. (3) Density and depth of CHI did not highly affect the CHI demand.

34	CHI density: Total premium/population	(1) Social health insurance density: Total premium from NCMS, UEBMI and URBMI/ population	(1) Social health insurance can drive the development of CHI with saving purpose but no effect on health protection-oriented CHI.

35	CHI Premium	(1) Total health expenditure (2) Proportion of elderly population (3) Total population (4) Chinese GDP (5) Balance of residents' RMB savings at the end of year (6) Education expenditure	(1) Total health expenditure, proportion of elderly population, balance of residents' RMB saving at the end of year could have a positive effect on CHI demand (2) GDP and education expenditure did not have significant effect on CHI demand.

## Abbreviations

CHI: Commercial health insurance.
